# Reconstruction of incomplete X-ray diffraction pole figures of oligocrystalline materials using deep learning

**DOI:** 10.1038/s41598-023-31580-1

**Published:** 2023-04-03

**Authors:** David Meier, Rishan Ragunathan, Sebastian Degener, Alexander Liehr, Malte Vollmer, Thomas Niendorf, Bernhard Sick

**Affiliations:** 1 Optik und Strahlrohre, Helmholtz-Zentrum Berlin für Materialien und Energie GmbH, Hahn-Meitner-Platz 1, 14109 Berlin, Germany; 2 Intelligent Embedded Systems, University of Kassel, Wilhelmshöher Allee 73, 34121 Kassel, Hessen Germany; 3 Institute of Materials Engineering, Metallic Materials, University of Kassel, Mönchebergstraße 3, 34125 Kassel, Hessen Germany

**Keywords:** Characterization and analytical techniques, Imaging techniques, Computational methods

## Abstract

X-ray diffraction crystallography allows non-destructive examination of crystal structures. Furthermore, it has low requirements regarding surface preparation, especially compared to electron backscatter diffraction. However, up to now, X-ray diffraction has been highly time-consuming in standard laboratory conditions since intensities on multiple lattice planes have to be recorded by rotating and tilting. Furthermore, examining oligocrystalline materials is challenging due to the limited number of diffraction spots. Moreover, commonly used evaluation methods for crystallographic orientation analysis need multiple lattice planes for a reliable pole figure reconstruction. In this article, we propose a deep-learning-based method for oligocrystalline specimens, i.e., specimens with up to three grains of arbitrary crystal orientations. Our approach allows faster experimentation due to accurate reconstructions of pole figure regions, which we did not probe experimentally. In contrast to other methods, the pole figure is reconstructed based on only a single incomplete pole figure. To speed up the development of our proposed method and for usage in other machine learning algorithms, we introduce a GPU-based simulation for data generation. Furthermore, we present a pole widths standardization technique using a custom deep learning architecture that makes algorithms more robust against influences from the experiment setup and material.

## Introduction

The relevance of characterizing coarse-grained structures and their orientations has increased considerably in recent years since coarse-grained structures tend to have anisotropic, i.e., direction-dependent, properties. These properties are very interesting for many materials being promising for industrial applications. As an example, the orientation of grains has a strong influence on the behavior of shape memory alloys (SMAs), e.g., on the transformation strain of singlecrystalline SMAs^1–4^ or oligocrystalline SMAs^5–9^. Moreover, additive manufacturing often promotes the evolution of coarse-grained columnar microstructures with a strong texture due to the specific local temperature history at each spot of the specimen during processing. Such microstructures can significantly affect the properties. Therefore, the examination of the properties of these promising materials is essential.

Up to now, it has been difficult and time-consuming to examine such microstructures, since excellent surface qualities are required for techniques such as electron backscatter diffraction, where the size of the probed specimen is limited. In addition, it cannot be ruled out that the complex specimen preparation does not already have an effect on the area to be examined, e.g., by stress-induced solid state transformation. By using X-ray diffraction, large specimens can be investigated, and the requirements for the specimen surface are comparatively low.

If arranged in an atomic lattice, multiple atoms will scatter the X-rays. Even if most scattered radiation erases due to negative interference, some rays add constructively in only few directions. We can determine these directions by using Bragg’s law^10^:

1 $$\begin{aligned} n\lambda = 2d \sin {\theta }, \end{aligned}$$

where *n* is the reflection order as an integer, $\lambda$ the wavelength of the X-ray radiation, $\theta$ the angle of incidence, and *d* the distance between *hkl* lattice planes, while *h*, *k,* and *l* represent the Miller indices.

**Figure 1:**
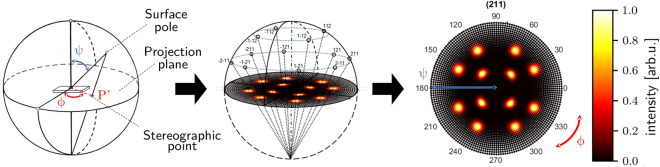
Schematic highlighting the link between rotation angles in the X-ray diffraction setup and pole plots. The surface of the sphere on the left can be imagined as spanned by a vector from the center of the sphere, tilted by all possible angles $\psi$ and rotated by all possible angles $\phi$. Any measured point with tilt $\psi$ and rotation $\phi$ is on this surface, we show an exemplary point labeled with surface pole. To generate a pole plot from this sphere, we draw a vector from the surface pole to the south pole. The intersection of this vector with the projection plane is the stereographic point, labeled with $P'$. The projection plane is the plane dividing the upper and lower halves of the sphere. We plot the intensity of the measured point at the intersection point $P'$, denoted with stereographic point. In the center plot, we show the projection of all accessible stereographic points of the lattice plane 211 for a single crystal. On the right is an example of a pole plot for this lattice plane. The intensity in the plot is the min-max normalized intensity of diffraction measured by the detector.

The signal of the diffracted X-rays is measured by a detector, which is placed at a diffraction angle $2 \theta$ to the X-ray source. We tilt and rotate the specimen while recording consecutive intensities. Thereby, we can record characteristic diffraction patterns^11^. We will denote the tilt angle with $\psi$ and the rotation angle with $\phi$. Thus, it is possible to analyze the presence of various diffraction peaks to evaluate, for example, lattice parameters, prevailing phases, internal stresses, or present grain orientations. In the following, we will focus on the analysis of grain orientations, and we will name this resulting image a pole figure or pole plot. The pole plots are always stereographic projections of the distributed intensity of crystal orientations for crystallographic lattice planes. In Fig. 1 we show how these pole plots are generated from the measured diffracted intensities at the respective tilt and rotation angles. We will refer to the two-dimensional Gaussians visible in the pole plot as poles, their size in $\phi$ and $\psi$ direction as pole widths.

Despite the above-mentioned advantages of X-ray diffraction, it is very time-consuming in the laboratory setup due to the low yield of photons in laboratory X-ray sources. Thus, high exposure times are required to get sufficient intensities. A complete measurement with the maximum possible tilt angle and measurement of three recorded *hkl* lattice planes requires about 10–30 h in a laboratory setup depending on optics, diffraction setup, and measurement strategies. This procedure includes the measurement outside of a diffraction peak, i.e., background intensity, for peak intensity correction amending the background and defocussing effect. Moreover, we must choose the size of the grid of the crystallographic hemisphere appropriately. In particular, for sharp crystallographic textures or oligocrystalline specimens, a standard measurement grid with $5^\circ$ or a continuous intensity detection is not sufficient. However, using a more detailed measurement grid increases the measurement time^11^.

A reduction of the measurement time is, therefore, highly desirable. This reduction can be achieved, for example, by decreasing the maximum recorded tilt angle, since pole figures contain redundant information. We will denote this maximum tilt angle with $\psi _{\textrm{max}}$. However, up to now, it is unknown which pole figure coverage ranges provide which level of precision. Therefore, we want to identify quantitatively which maximum tilt angle is required for which level of reconstruction error in our proposed method.

There is even a maximum physically reasonable tilt angle due to the diffraction setup and incidence angle, resulting in reduced intensity related to the increased attenuation in the elongated irradiation path and the defocussing effect^11,12^. This measurement limitation requires accurate pole plot reconstruction algorithms, the calculation of orientation distributions, or measurement adaptations to provide consistent and complete pole plots^13,14^.

An additional advantage of reconstructions of unmeasured pole plot regions is that they can provide the experimenter with decision-making support whether a running measurement should be continued. For example, the experimenter can check if a predicted pole spot was hit or if the measurement should be continued.

A challenge that arises is pole broadening induced by microstructure variations or thermal and experimental setup changes. Only the positions are relevant for the analysis of crystal orientations, and different pole sizes complicate further processing by algorithms. Thus, a solution for standardizing pole widths is desirable to make subsequent processing algorithms more robust against disturbances.

For the crystal orientation analysis, the Laue method is usually applied for singlecrystalline specimens to detect a high number of poles of a single grain by using polychromatic radiation and an area detector with a fixed diffraction setup. By this, the diffracted X-Rays can be recorded for various lattice planes without a specimen movement according to the wide range of wavelengths and results in characteristic patterns. However, for imperfect crystals or different grains, an analysis of the overlapping high number of diffraction spots is not feasible^11,15^. For a polycrystalline, textured specimen incompletely measured pole figures can usually be completed mathematically on the basis of orientation density functions (ODF)^13^. The idea of this approach is to determine the ODF as precisely as possible based on the available data. Missing information for determining an ODF can be supplemented by measurements on further *hkl* lattice planes in the same measurement range and same specimen volume. The reconstruction error thus can be decreased by high data quality and a higher number of different *hkl* lattice planes, which also result in additional measurement effort. Pole figure inversion is essential for determining the ODF. It can be solved, for example, by the so-called component method^16,17^, direct methods like the WIMV algorithm (named after its authors Williams, Imhof, Matthies and Vinel)^18–20^, or a series expansion method^11,13,21,22^. Fourier transformation methods are commonly used to solve a series expansion. We can use special evaluation programs for this approach, such as the MATLAB-based program MTEX^23^. Using the ODF, the reconstruction of a pole figure of a specimen volume with a low number of grains with highly differing orientations is error-prone because the harmonic method requires smooth functions. Thus, it cannot avoid artifacts like ghost peaks or negative values due to the complexity of the algorithms^13^. Especially sharp crystallographic textures or a few different grains in the specimen volume create physically incorrect poles or oscillations in the calculated pole figure. Although extensions and adaptions for the harmonic method overcome some of these issues^24–28^, a correct determination of the ODF for oligocrystalline material remains challenging. By applying the direct method WIMV on data with the normalized intensity of incomplete pole figures, the mentioned issues are solved with the method itself. However, direct methods have a higher susceptibility to data noise and disturbed data^13^, which is very likely in a laboratory setup due to the low primary intensity. With lower grain counts, though, the results are more reliable also with the direct methods because of decreased ambiguity of the pole plots. Furthermore, pole figure data of at least three *hkl* lattice planes are needed to achieve good results. Both increase the experiment time and measurement effort. Therefore, we introduce a method that only needs partly measured data of only one pole figure of material with a few differently oriented grains. Our presented approach works reliably and extremely fast to reconstruct the complete pole figure, even during the measurement process. By this, we reduce the data preprocessing steps and make our method robust against instrumental influences on the data.

The main advances provided by the method proposed in the present study are:

### Fast pole plot simulation

Since we use machine learning for subsequent processing, we need a high number of data samples for training. To provide the required data amount, we used a simulation that maps one or multiple grain orientations to a resulting pole plot. The presented Graphics Processing Unit (GPU) based implementation enables parallel and online creation of pole plots. It is usable in diverse optimization scenarios or for creating huge machine learning datasets. It even allows infinite online creation of training data. Furthermore, it can be used in brute-force or other global optimization algorithms for applications in unsolved problems, e.g., grain orientation determination from pole plots.

### Pole widths standardization

To make our method applicable to data stemming from specific microstructures and measurement facilities, we propose a custom deep learning architecture, that standardizes pole widths. It provides fast standardization of pole plots on GPU and can standardize many pole plots in parallel. Furthermore, it is robust against noisy inputs, i.e., errors of the pole widths standardization do not increase significantly by adding high levels of Gaussian noise to the input pole plots.

### Faster experimentation due to accurate reconstructions

With our proposed deep learning method, we can reconstruct pole figure regions, which we did not probe experimentally. Our approach works also on oligocrystalline specimens with only few grains in the probed area. By comparing the complete data with reconstructed data, we can determine the error of the prediction for specific sizes of missing parts of the pole plot. Thus, we can give the experimenter decision-making support on how much measurement time can be saved without severe information loss. We also present an extension of our approach, which allows providing the algorithm’s uncertainty of the reconstructions in a spatial resolution.

## Results and discussion

This section shows the results of our proposed method and discusses its accuracy and applicability.

### Pole Widths Standardization

**Figure 2:**
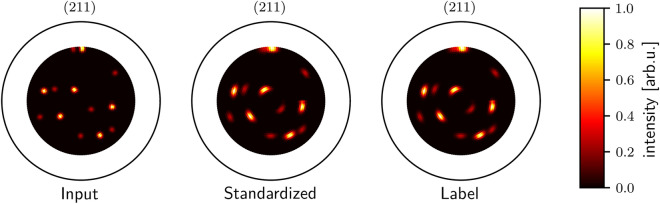
Exemplary result of standardization for a 211 lattice plane pole plot with three visible grains. On the left, we show an input plot with $\sigma _\phi =1$ and $\sigma _\psi =2$. In the middle, we plot the standardized output with normalized pole widths of $\sigma _\phi =\sigma _\psi =2$. There is no noticeable visual difference between the standardized output and the label, except for a slightly brighter background. All pole plots are min-max normalized.

We developed the evaluation strategy presented here to be used more extensively in future applications. As a metric of the error of pole plot standardization, we use the mean squared error (MSE) over the standardized intensity values of 100,000 randomly generated pole plots with $\psi _{\textrm{max}}$. We consistently average the deviation per intensity value on normalized pole plots. Thus the MSE can be viewed as a quadratic relative deviation. We used one to three-grain pole plots for evaluation. For training and evaluation, the pole widths are chosen randomly, with the pole width of the rotation angle $\sigma _\phi \in [0.5, 2.5]$ and tilt angle $\sigma _\psi \in [0.5,5]$. We chose these intervals to cover the most common experiment setups and materials. In Table 1 we present the MSE with increasing $\psi _{\textrm{max}}$ values. Experiments of $\psi _{\textrm{max}}=70^\circ$ have a higher MSE compared to $\psi _{\textrm{max}}=60^\circ$. The reason for this increase might be that the pole widths in outer regions are harder to standardize since correlations between the shapes of poles are more complex there. Since we only take the mean over the predicted intensities, they significantly impact the MSE. For reference, we calculated the MSE of 100,000 blank pole plots compared to simulated pole figures of equally distributed grain orientations. This procedure simulates an empty output of the standardization network and can be used to assess the applicability of the MSE as a metric. The MSE of the reconstructions is smaller than the MSE of the corresponding references. We show a sample evaluation in Fig. 2. For the three-grain pole plots, there is no difference perceivable between the standardized output and the label, except for a slightly brighter background. By label, we mean the pole plot with the same grain orientations but simulated with the targeted normalized pole widths.

**Table 1:** MSEs of the pole widths standardization approximation for $\psi _{\textrm{max}}$ of $40^\circ$, $50^\circ$, $60^\circ$, $70^\circ$. Pole plots contain grains with equally distributed grain numbers in the range of 1–3 grains. Lower values of MSE indicate better approximation. The reference values constitute the MSE when the samples are compared with a completely black pole plot, i.e., when all intensity values are 0. We calculated the MSE for 100,000 simulated pole plots of equally distributed grain orientations per $\psi _{\textrm{max}}$. This reference pretends that the standardization was a blank pole plot to judge the shown MSE values. The MSE of the reconstructions is far smaller than the MSE of the corresponding references.

$\psi _{\textrm{max}}\ ({}^{\circ })$	MSE	Reference
40	0.0011	0.0162
50	0.0007	0.0155
60	0.0003	0.0149
70	0.0006	0.0143

We applied noise to the generated pole plots to show that our approach is robust against disturbances. The noisy data are not drawn from the same distribution as the training data, since we did the training without noise. We generated noisy test data like shown in the following equation:

2 $$\begin{aligned} I_{noise} = I + \epsilon \text {, with} ~ \epsilon \sim \mathcal {N}\left( {\textbf {0}}, p \cdot \sigma \left( I\right) \right) \end{aligned}$$

We denote the resulting intensity, including noise with $I_{noise}$ and the initial intensity from the simulation, with *I*. We draw $\epsilon$ from a normal distribution with mean ${\textbf {0}}$ and standard deviation $p \cdot \sigma {(I)}$, where *p* denotes the level of noise and $\sigma {(I)}$ the standard deviation of the intensities. The results are shown in Table 2. Despite the added noise, the MSE of the standardization does not increase considerably. That proves that our standardization model still works with normally distributed noise.

**Table 2:** MSEs of the pole widths standardization approximation for $\psi _{\textrm{max}}$ of $50^\circ$ and $60^\circ$ and different amounts of noise (without, $10\%$, and $20\%$ noise). The simulated pole plots contained 1, 2, or 3 grains equally distributed. Lower values of MSE indicate better approximation. As to be expected, the pole widths standardization approximation is less accurate in case of a higher level of noise. The references were calculated the same way as in Table 1.

$\psi _{\textrm{max}}\ ({}^{\circ })$	*p*	MSE	Reference
50	0	0.0007	0.0155
50	0.1	0.0007	0.0156
50	0.2	0.0007	0.0156
60	0	0.0003	0.0149
60	0.1	0.0003	0.0149
60	0.2	0.0003	0.0149

### Reconstruction

**Figure 3:**
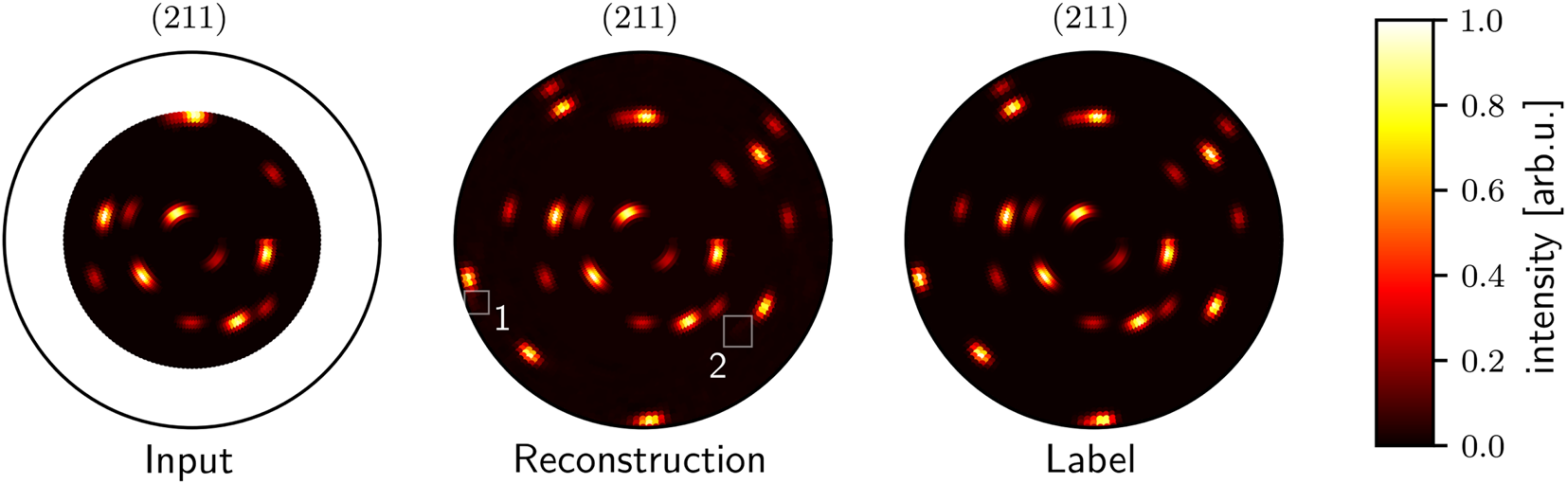
Reconstruction of missing tilt angle intensities for a 211 lattice plane pole plot with three visible grains. On the left, the simulated cropped input with $\psi _{\textrm{max}}=60^\circ$ is shown. The plot in the middle is the output of the reconstruction network. On the right is the entire label as output by the simulation. In the reconstruction plot, it is visible in the rectangle denoted with 1 that the reconstruction network overdraws a pole. In the area labeled with 2, the reconstruction slightly indicates some poles that do not exist in the label. Despite these artifacts, the overall reconstruction appears accurate. Furthermore, the background of the reconstruction appears to be slightly brighter compared to the label. All pole plots are min-max normalized.

**Figure 4:**
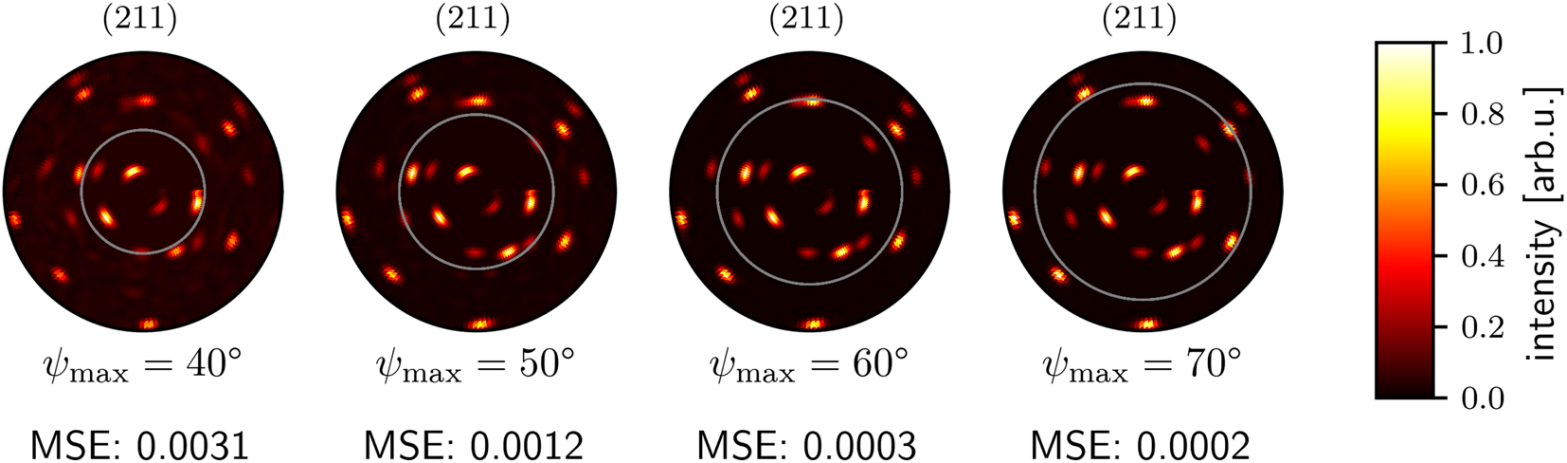
Comparison of reconstructions with different maximum tilt angles for the same 211 lattice plane pole plot as in Fig. 3. We plot the input sample inside of the gray circular boundary line. Outside this gray boundary, we plot the reconstruction by the corresponding reconstruction network. It is visible that given an input pole plot with $\psi _{\textrm{max}}=40^\circ$, many artifacts and false poles are reconstructed. The reconstruction with $\psi _{\textrm{max}}=50^\circ$ shows considerably less background noise. The further steps are without noticeable differences compared to the label. For the label, please refer to Fig. 3. All pole plots are min-max normalized.

We also use the MSE as metric for comparing the predicted intensities with the real intensity values from the complete pole plot of the reconstruction. We generated plots of 100,000 equally distributed grain orientations with the simulation containing one to three grains. Higher grain counts are possible but increase training time and require adaptations in the network architecture. Thus, they are not examined in this article. The randomly generated samples for testing the reconstruction were not used for training the neural network. We show the achieved MSE values in Table 3. As expected, reconstruction error increases with the number of grains since the algorithm needs to reconstruct more poles with a higher discrepancy in their intensity. It is challenging for the network to reconstruct poles with low intensities since errors and thus gradients are low. Neural networks are trained by updating their weights according to the gradients of the errors. Thus, training is hard if low gradients are occurring. In Table 4 we list the relative improvements of MSEs that the algorithm can achieve by increasing $\psi _{\textrm{max}}$ by $10^\circ$. In other words, we determine the error difference for measuring an additional tilt angle range of $10^\circ$. It is noticeable that on the one hand, the error decreases most for the step from $\psi _{\textrm{max}}$ of $40^\circ$ to $50^\circ$, i.e., by $46.81\%$. On the other hand, the error seems to even increase for the step from $\psi _{\textrm{max}}$ of $60^\circ$ to $70^\circ$. This behavior is induced by the same reason as in the seemingly worse pole standardization, because outer regions contain more complex structures. Thus, the MSE increases despite better reconstruction since only these regions are considered for calculating the calculation of the error. Furthermore, a smaller number of grains generally profits more in terms of the achieved approximation error. One conclusion we can draw is that measuring up to an angle of $\psi _{\textrm{max}}=60^\circ$ still notably decreases the MSE of the reconstruction. For higher values, the gain is less considerable. We depict these results in Fig. 3. Despite some slightly noticeable artifacts, the overall reconstruction appears visibly accurate. These artifacts could be induced by the imprecision of the average pooling layer that is required to keep the MLP-Decoder computationally feasible. Since these artifacts could be mistaken for additional grains in subsequent algorithms, they should be removed by choosing an appropriate threshold. In Fig. 4 we compare reconstructions with different maximum tilt angles. While the reconstruction quality from $\psi _{\textrm{max}}=40^\circ$ to $50^\circ$ increases remarkably, the further steps to $60^\circ$ and $70^\circ$ increase the reconstruction quality only slightly.

**Table 3:** MSEs for reconstruction with $\psi _{\textrm{max}}$ from $40^\circ$ to $70^\circ$ and different numbers of grains. Lower values of MSE indicate better approximation. As to be expected, the error of reconstruction increases with the number of grains and decreases with higher $\psi _{\textrm{max}}$.

$\psi _{\textrm{max}} [^\circ ]$	1 Grain	2 Grains	3 Grains	Mean
40	0.0036	0.0047	0.0058	0.0047
50	0.0013	0.0026	0.0036	0.0025
60	0.0008	0.0018	0.0026	0.0017
70	0.0012	0.0019	0.0026	0.0019

**Table 4:** Relative difference of MSEs for reconstructions when adding $10 ^\circ$ for $\psi _{\textrm{max}}$ of $40^\circ$, $50^\circ$ and $60^\circ$ for pole plots with different numbers of grains.

	1 Grain (%)	2 Grains (%)	3 Grains (%)	Mean (%)
$\epsilon _{40^\circ \rightarrow 50^\circ }$	− 63.89	− 44.68	− 37.93	− 46.81
$\epsilon _{50^\circ \rightarrow 60^\circ }$	− 38.46	− 30.77	− 27.78	− 32.00
$\epsilon _{60^\circ \rightarrow 70^\circ }$	+ 50.00	+ 5.56	± 0.00	+ 11.76

### Uncertainty determination of reconstruction

**Figure 5:**
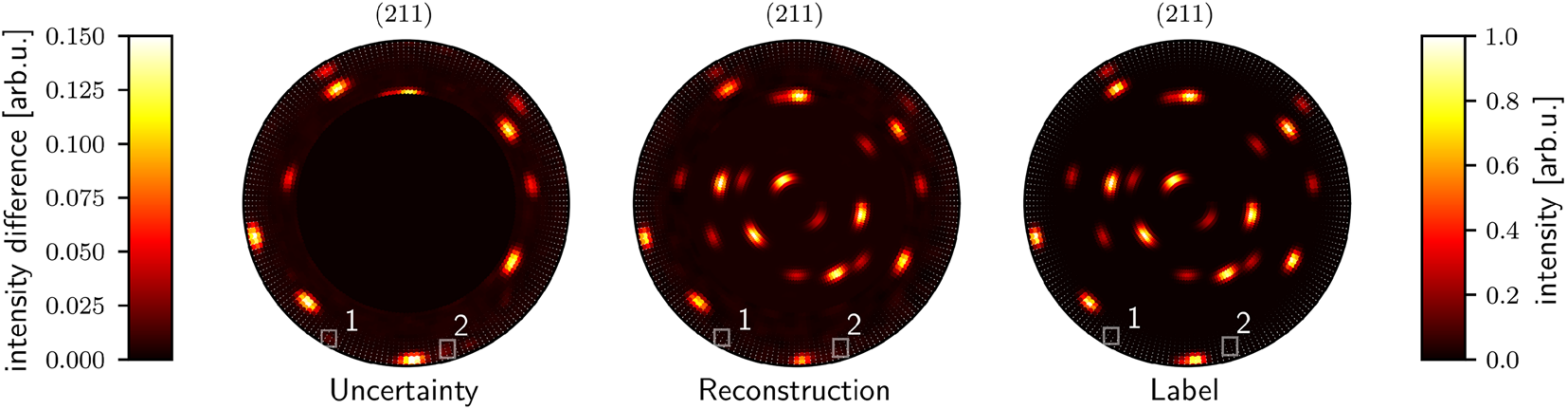
Uncertainty of reconstruction for a 211 lattice plane pole plot with $\psi _{\textrm{max}} = 60^\circ$ and three visible grains. The left plot shows the standard deviation of outputs of the Monte Carlo dropout network, which we denote with the uncertainty of the model. We used 1000 repetitions of the same input to get sufficient coverage of the output distribution of the dropout network. The input is similar to Fig. 3. The left colormap describes the uncertainty. The right colormap describes the reconstruction and label. All pole plots are min-max normalized, except for the uncertainty plot, which is the standard deviation of min-max normalized pole plots. The area labeled with 1 contains a pole visible in the reconstruction but no pole in the label. Thus, a higher value in the uncertainty plot is visible. The rectangle denoted with 2 contains a higher value for uncertainty. However, there is no pole neither on the reconstruction nor on the label. That means that in some reconstructions a false pole is drawn at this point.

To extend our approach, we want to measure the reconstruction error and know at which measurement orientations the resulting algorithm is uncertain of the approximation of the intensity. We do this by using Monte Carlo dropout. We show the results of the uncertainty determination in Fig. 5. Obviously, the model is especially uncertain in the regions where the reconstruction error is high, i.e., the pole intensity is not entirely correct, or a non-existing pole was reconstructed. The MSE is 0.0043 per predicted intensity value for $\psi _{\textrm{max}} = 60^\circ$. Please note that the reconstruction quality is decreased due to the use of dropout in comparison with the model used in Fig. 3.

### Real-world sample

**Figure 6:**
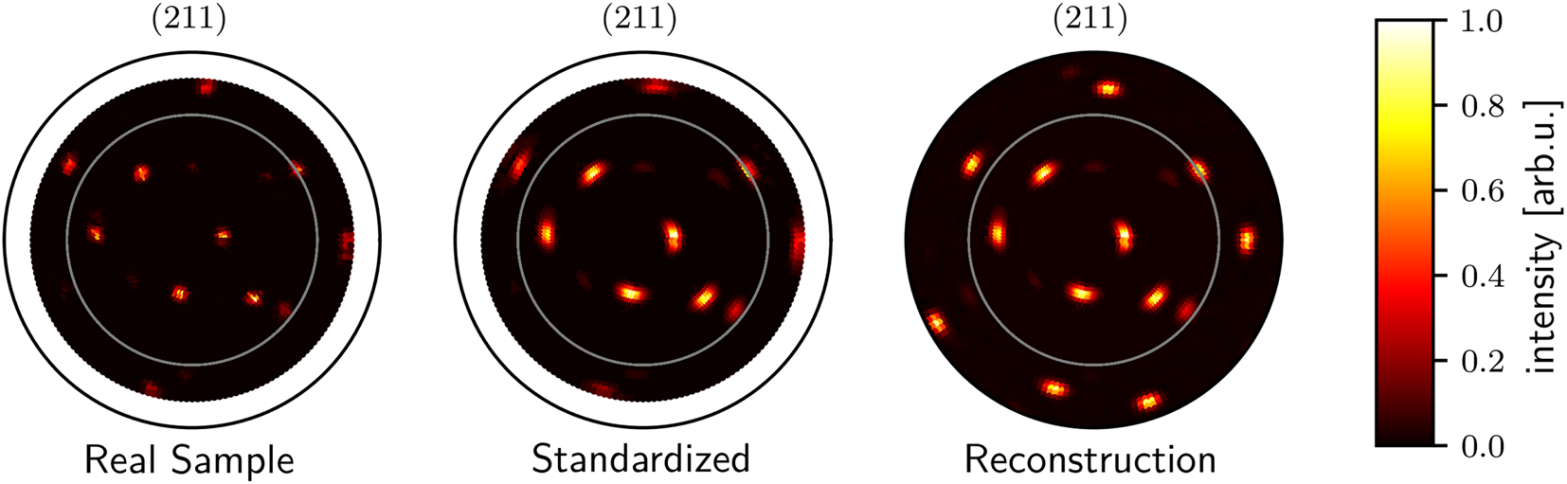
Reconstruction of a 211 lattice plane pole figure of a Fe–Mn–Al–Ni–Ti specimen. The label from the real pole plot was recorded up to $\psi _{\textrm{max}}=76^\circ$. However, we input only the part of the real pole figure up to $\psi _{\textrm{max}}=60^\circ$, so we can use the residual measurement as verification of our reconstruction. In the real sample plot, we plot the input sample inside of the gray circular boundary line and outside of the gray line we plot the rest of the real sample that was still recorded but not shown to the neural network. In standardized we also plot the real sample but after pole plot standardization. In reconstruction, we plot the standardized version of the real data within the gray boundary which is the input for the reconstruction network. Outside of the boundary line, we plot the complete recorded data in the input with $\psi _{\textrm{max}}=60^\circ$. The reconstruction network can restore the position of all poles, even the poles with low intensity. All pole plots are min–max normalized.

To verify the applicability of our method in real-world settings, we reconstruct the data of a real-world sample. To properly evaluate our method, we use a real specimen that we measured up to $\psi _{\textrm{max}} = 76^\circ$. For a pole figure of the 211 lattice plane with the density of measuring points shown in Fig. 6 a time of 228 min was needed without specimen mounting. With the before mentioned methods, at least three lattice planes are required for a reliable reconstruction of grain orientations. We simulate a smaller measured pole plot by omitting the intensities after $\psi = 60^\circ$, and, thus, only needed 180 min measuring time. In comparison to the direct methods, we are able to save 504 min (~74%) test time for reconstruction due to the smaller number of required lattice planes to be measured and the shorter measuring time per pole plot. By reconstructing and comparing the missing poles, we can see if the poles get reconstructed properly. The results are shown in Fig. 6. The figure shows that the reconstruction network can restore all positions correctly, even poles with low intensity. The effect that outer intensities are less distinct because of the varying radiation path of the real sample and in the standardized pole plot. The reconstruction network abstracts from this phenomenon since the training data did not contain this effect and shows all poles in full intensity. The pole widths standardization does not abstract from this phenomenon because it only learns to standardize pole widths and does not modify the intensities of the poles. To statistically prove that our proposed method works in different real-world scenarios, we require evaluation with more specimens and materials in follow-up studies.

Our approach allows faster experimentation with accurate approximations. The entire pipeline is evaluated in less than 90 ms on an Intel i5-7200U CPU. We have verified that our approach works using simulation with entire labels and real-world plots using limited but meaningful labels. The differences between simulation and reality are minor and do not affect the applicability of our method since our approach is robust against minor disturbances. To conclude, we provide a compound solution that allows fast pole width standardization and reconstruction of missing tilt angles in coarse-grained materials.

## Conclusion and outlook

Our method provides fast and efficient standardization of pole widths enabling various algorithms to operate even though trained or tested considering only a single constant pole width. Thereby, the development and training of machine learning methods can be supported, and broader applicability for different microstructures, materials, and measurement facilities, which might result in changed pole widths, can be reached. Furthermore, we provide an algorithm for reconstructions of incomplete pole plots with limited tilt angles for multiple grains. By examining the error differences, we can determine which pole figure coverage the algorithm can achieve at a selected maximum tilt angle. Thus, we give the experimenter decision-making support in measurement time versus reconstruction error reduction considerations. Furthermore, the experimenter gets an approximated preview of the entire plot and can decide very early if the examined spot is interesting, and thus the measurement should be continued. Due to the lack of a sufficient amount of real material samples, all our proposed machine learning algorithms would be insolvable without leveraging knowledge from the presented simulation.

However, many modifications of our approach are possible. For example, we could examine differences in achievable reconstruction error with maximum rotation angles or both tilt and rotation angles to find optimal measurement conditions for specific microstructures, specimen geometries, and measurement setups. Another interesting question is whether the combination of different lattice planes is beneficial in a measurement time versus reconstruction error consideration. This point of view can be highly advantageous for energy-dispersive measurements as in that case all lattice planes are measured and, thus, containing redundant information. A lot of measurement time could be saved by requiring smaller maximum tilt angles. A promising possibility for faster training and more accurate algorithms would be investigating a different error measure than the MSE. The MSE is not optimal since it does not correctly reproduce the actual error distance between two solutions since a pole plot is a stereographic projection. That means all boundaries of the pole plot are connected with the opposite side of the projection. The MSE does not map this relation since it does not recognize if a pole is near the boundary. Thus, the resulting pole plot with a pole on the opposite boundary is a similar solution. Furthermore, we cannot distinguish between low background noise in the reconstruction and incorrectly predicted poles. For example, we could use the Wasserstein metric, also known as Earth Movers’ distance, which matches the perceptual similarity better than other metrics^29^. However, this metric has the disadvantages of being harder to interpret and having higher computational times.

We will extend all experiments to higher grain counts and use the results of the uncertainty determination for an active experimentation approach where the X-ray diffraction device could actively examine regions with high uncertainty, as proposed in^30^. Please note that this measure for uncertainty does not cover ambiguity of the dataset mappings, e.g., if there are multiple possible mappings of the outer approximated area of the pole plot fitting to one input. We require methods to learn probability distributions of possible outputs like variational autoencoders or invertible neural networks. We could use the gained knowledge to actively examine regions of pole plots with high ambiguity to reduce approximation reconstruction error significantly at a low increase of measurement time.

We are developing a brute-force method for grain orientation determination which depend on our proposed fast simulation and pole widths standardization. However, a method for determining the number of grains and standardizing the intensities of different grains on one pole plot is required for universal applicability in pole figures.

## Method

### Simulation

For training the machine learning methods we developed a simulation that creates a pole figure plot as output for a given orientation. As input parameters, the grain orientation, the *hkl* lattice values, the minimum and maximum desired tilt angle, and the pole widths $\sigma _{\phi }$ and $\sigma _{\psi }$ can be specified. We chose quaternions which are vectors consisting of four values as input format since they are not ambiguous^31^, unlike the representation in three Euler angles. The direct translation from a quaternion to an Euler angle representation is trivial. The source code is publicly available (https://git.ies.uni-kassel.de/digiwerk/pole-plots/pole-plot-simulation). It runs entirely on GPU and thus enables a considerable performance boost for the presented pole widths standardization and reconstruction methods.

To simulate oligocrystalline structures with up to thousands of grains, we generate multiple grains randomly and calculate a weighted sum of these grains. We draw the weights from a Dirichlet distribution with $\alpha = {\textbf {1}}$, i.e., the weights sum up to 1, and we distribute them uniformly random. We chose a uniform distribution of the weights to make as least assumptions as possible about the distribution of occurring weights.

All simulations used for training and the measurements for evaluation were made for 211 lattice planes due to the high number of poles for the low number of grains and their good peak quality in the performed laboratory experiments. The measurement grid size of the pole figures was $2^\circ$ for tilting and rotation axes, with a counting time for each specimen orientation of 0.5 s. Moreover, we always use one to three grains to simulate structures, which is common for X-ray diffraction measurements of oligocrystalline specimens.

For a single lattice plane and a single grain, with our simulation on an Nvidia Tesla A100 GPU, we can calculate about 100,000 pole plots per second. Because of the data generation during training, the simulation enables us to use more than 250 million samples for training since we neither store them on a hard drive nor need to transfer them to video memory.

There are some simplifications and limitations of the simulation. We do not consider the intensity decrease caused by the varying radiation path in the specimen material and the defocussing for different inclination angles in all generated plots. We could diminish this issue by correcting the data by subtracting a background noise plot and defocussing pole plot as preprocessing steps. These plots are generated by a further measurement of the background intensity outside an interference position on the same material specimen. Thus, it contains the static noise and background intensity variation induced by the experimental measurement setup and material. In addition to measurement-based corrections, there are approaches to calculating correction factors^32^. Subsequently, it is possible to calculate intensity values as multiples of a random distribution (MRD) of the measured intensities to enhance the comparability and interpretation of various pole plots^11^. This procedure is of particular interest for quantitative texture analysis but does not highly affect the data of oligocrystalline structures since we usually have high pole intensities compared to the background noise, and thus the absolute intensities are not that important. For this reason, we omit these corrections to save measurement time and preprocessing steps.

Moreover, compared to the simulation, there might be a lack of measured poles in real data related to the gauge volume variation by specimen rotation and tilting. Depending on the grain locations and their dimensions regarding the irradiated specimen volume, this effect can be relevant. This issue is more pronounced for small diffraction angles with an elliptic gauge volume and grain sizes in the order of the used gauge volume. However, this is not relevant for the present study since we only consider grain counts of up to three huge grains, which are constantly covered by the gauge volume. Furthermore, our used lattice plane 211 has relatively high incidence angles, and thus this issue is further reduced. Thus, the simulation includes all accessible poles, and we ignore the effect of lacking poles due to the gauge volume.

### Error measure

We use the MSE per intensity value for all evaluations in this article as a measure of error. The MSE fulfills precisely the desired properties: It puts less weight on minor differences in the background intensity and weights errors in the relevant areas, the poles with high intensity, very high. This approach can cause a bias for polycrystalline materials because the MSE underrates errors in low-intensity regions, but we do not survey these in this article. Other error measures, like the mean absolute error (MAE), turned out to be relatively poor since our visibly well-approximated pole figures had similar errors to completely black (background intensity) images. This phenomenon occurs because minor deviations in the dark areas with no or less intensity accumulate. In contrast to the analysis of polycrystalline materials, these dark areas contain only background noise and are thus not of interest in our present study.

### Pole widths standardization

For standardizing pole widths, we train a neural network that gets a pole plot as input and outputs a pole plot with poles of similar widths. The input of the network is statistically standardized, i.e., the input intensities per image have a mean of 1 and a variance of 0. We use a neural network architecture similar to the U-Net autoencoder proposed by^33^ trained with randomly generated pole plots by the simulation.

An autoencoder is a neural network consisting of two parts: The encoder is composed of several layers with decreasing size. The last layer of this encoder is called bottleneck, and its neuron count is called bottleneck size. The second part, the decoder, typically has the same amount of layers but with symmetrically increasing layer sizes. Usually, one trains an autoencoder to reconstruct the input data precisely. This procedure allows us to learn a compressed representation of the input data using the encoder and enables the decoder to unfold the data. In our case, we use the autoencoder to standardize the pole width. We train this behavior by generating samples consisting of two parts:

1. The input part is a simulated pole plot with a random number of grains, orientations, and pole widths.
2. The label part is a pole plot generated with the same number of grains and the same orientations as their corresponding input, i.e., the poles are at the same positions. Only the pole widths differ; they are not randomly chosen but use the standardized $\sigma _\phi =2$ and $\sigma _\psi =2$.

By training the network to return the corresponding standardized label output to an input with random pole widths, we enforce it to learn how to standardize pole plots.

**Figure 7:**
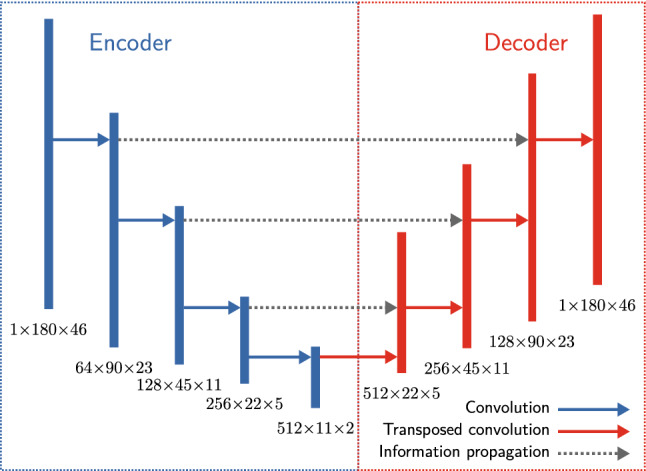
Basic U-Net-like architecture of the pole widths standardization algorithm. The numbers in the figure describe the dimensionality of the resulting tensors ($\textrm{depth}\times \textrm{height}\times \textrm{width}$) after applying the corresponding layer. The heights of the rectangles are arbitrary. The gray arrows depict the information propagation to a subsequent layer.

The U-Net architecture is an autoencoder using convolution layers, but it can pass some compressed information from the encoder to the decoder on the same level and thus often provides more accurate predictions. We depicted our U-Net-like architecture in Fig. 7, where the information propagation process is pictured with gray arrows. In our case, the U-Net-like architecture also outperformed conventional autoencoders in terms of standardization error.

The encoder of our U-Net-like architecture consists of four convolution layers. We assume basic knowledge of convolutional neural networks; for more information, consider^34^. We chose this architecture by evaluating many architectures and parameters and selected those with the least standardization error. The used filter size is $4\times 4$. We set the padding to 1 and stride to 2. The padding is the number of values added at the boundary of the input tensor. By this extension, the values close to the boundary of the tensor are weighted similarly to inner values in the resulting output. The stride defines how many steps the filter is shifted when it slides across the input tensor. The decoder uses four transposed convolution layers. A transposed convolution layer is an upsampling convolution, i.e., the output dimension is higher than the input dimension. After every convolution or transposed convolution layer, we apply the Mish activation function proposed in^35^. To give information from the encoder to the decoder, we concatenate every layer’s output with the corresponding layer’s input on the same level. Since the layer sizes do not fit due to odd and even numbers, we use bilinear interpolation to fit the layer sizes of the outputs of the transposed convolution layers to the corresponding encoder output tensors. Bilinear interpolation is an interpolation method that applies repeated interpolation to reduce dimensionality while sustaining the proportions of the tensor entries. We trained the U-Net-like neural network for 50,000 epochs with batch size 500. A batch is the number of samples trained simultaneously before the neural network weights get adapted. Per epoch, two batches are shown to the network. That means during training, we generated in total 50 million training samples. For training, we used the Adam optimization algorithm with learning rate $\eta = 10^{-6}$ and MSE as loss function. We selected the model with the least validation error for evaluation.

**Figure 8:**
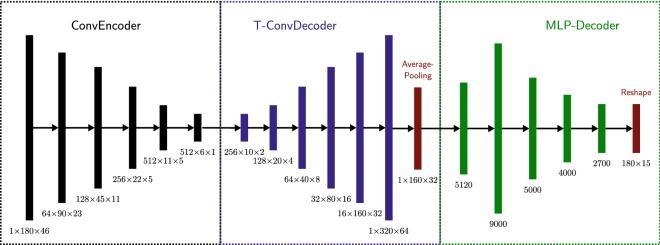
The basic reconstruction network architecture. It consists of three components: a convolutional encoder (ConvEncoder), transposed convolutional decoder (T-ConvDecoder), and a multilayer perceptron decoder (MLP-Decoder). The numbers show the dimension of output values per layer. We reshape the output of the MLP-Decoder to get the reconstructed unknown part of the pole plot for $\psi _{\textrm{max}} = 60^\circ$.

### Reconstruction

For reconstructing the unmeasured parts of a pole plot, we use a custom deep learning architecture that we will refer to as *reconstruction network*. It is designed to work with per image standardized input with $\psi _{\textrm{max}} \in \left\{ 40^\circ , 50^\circ , 60^\circ , 70^\circ \right\}$. Furthermore, the input data have to be peak width standardized. This preprocessing is done to ensure the applicability to measured data of different materials and different instrument broadening without a more extensive model training covering all possible peak widths. This circumstance would increase the required training data and training time by magnitudes. The authors also performed experiments focusing on learning peak position coordinates, but this led to convergence issues of the reconstruction networks.

The basic architecture is shown in Fig. 8. It consists of three components: a convolutional encoder (ConvEncoder), transposed convolutional decoder (T-ConvDecoder), and a multilayer perceptron decoder (MLP-Decoder). We use convolutional layers since they reduce the training time and memory consumption in comparison with fully connected MLPs. We utilize the ConvEncoder to bring the input pole plot to a low-dimensional representation. It comprises five convolution layers and calculates a bottleneck tensor size of $512 \times 5 \times 1$ ($\textrm{depth}\times \textrm{height}\times \textrm{width}$). After that, the T-ConvDecoder increases the dimensionality of the output to $1\times 320 \times 64$. An average pooling layer that calculates the means of a $2\times 2$ filter is subsequent. This average pooling layer reduces the dimension of the input for the subsequent MLP decoder. Thus, the input dimension for the MLP-Decoder is $1 \times 160 \times 32$. The MLP-Decoder consists of a five-layer fully connected perceptron that reconstructs the output intensities. We set up the transposed convolution layers similar to the encoder convolution layers. That means we set the filter to size $4\times 4$, padding to 1, and stride to 2. We provide the output tensor sizes and an overview of the architecture in Fig. 8. Except for the output layer, all layers use the Exponential Linear Unit (ELU) activation function. The output layer does not use any activation function. We apply a supervised learning strategy by the provision of simulation data with the plot cropped up to a defined $\psi _{\textrm{max}}$ as input and give it the unknown remaining plot as a label. We trained the reconstruction network for $\psi _{\textrm{max}}=60^\circ$ using the Adam optimization algorithm with learning rate $\eta =10^{-5}$ and MSE loss function. The network was trained for 50,000 epochs with batch sizes of 500, 1000, 1000, 1500 samples for the reconstruction networks with $\psi _{\textrm{max}}=40^\circ , 50^\circ , 60^\circ , 70^\circ$. Per epoch, two batches are shown to the network, i.e., for the model with $\psi _{\textrm{max}}=70^\circ$, 150 million artificial training samples were generated, and we chose the model with the least validation error for evaluation. We trained the networks for the other $\psi _{\textrm{max}}$ values with the same hyperparameters. The only exception is the network for $\psi _{\textrm{max}}=40^\circ$ where we adapted the learning rate to $\eta =10^{-4}$ since the resulting MSE on testing data was slightly smaller.

We determine the uncertainty by using *Monte Carlo dropout* with a dropout probability of $p=0.2$ in every layer except for the output layer. A dropout probability of $p=0.2$ means that in training and testing on average variable $20\%$ of the nodes are disabled. Nodes in subsequent layers connected to currently disabled nodes do not receive any signal from disabled nodes. This way, we can calculate the uncertainty per intensity by feeding the same input pole plot multiple times during testing and determining the different outputs’ mean.

We disabled the dropout in all other experiments to avoid a decrease in reconstruction quality. Please note that you can not infer the determined uncertainties to the reconstruction network without dropout, but we can use it for further applications, for example, for reducing the measurement time further at high reconstruction quality with active experimentation.

### Evaluation of real data

An evaluation of the proposed method is done on real data gained by pole figure measurements on a 300 mm long Fe-Mn-Al-Ni-Ti bar with a diameter of 6.3 mm consisting of two abnormally grown grains. The shape memory alloy is a promising candidate for large-scale applications due to the low costs of alloying elements and the potential use of established processing routes from the steel industry. Moreover, the cyclic heat treatment can lead to the formation of subgrain structures and, therefore, to broad peak intensities, which is an additional challenge for the approach. Therefore, it is well suited as a real-world example. Such coarse grain structure was obtained by a cyclic heat treatment, which leads to abnormal grain growth and grain size of several millimeters^36–39^. These two grains have been examined at the grain boundary of the Fe-Mn-Al-Ni-Ti bar for this evaluation. The investigated lattice plane 211 of the present body-centered cubic (BCC) phase was measured using a cobalt anode at $2\theta =98^\circ$ on the diffractometer Seifert XRD 3003 Micro operated at 40 kV and 30 mA, equipped with a monochromator in the secondary beam paths and a polycapillary with a beam size of 3 mm in diameter in the primary beam path.

## Data Availability

All simulated data can be generated with the presented simulation. The real data sample is available in the data repository of University of Kassel: https://doi.org/10.48662/daks-14.

## Code availability

The code for the simulation and evaluation is available at Gitlab of University of Kassel: https://git.ies.uni-kassel.de/digiwerk/pole-plots.
